# Background Load Denoising across Complex Load Based on Generative Adversarial Network to Enhance Load Identification

**DOI:** 10.3390/s20195674

**Published:** 2020-10-05

**Authors:** Afifatul Mukaroh, Thi-Thu-Huong Le, Howon Kim

**Affiliations:** 1 School of Computer Science and Engineering, Pusan National University, Busan 609735, Korea; afifatul.mukaroh@pusan.ac.kr; 2 IoT Research Center, Pusan National University, Busan 609735, Korea; lehuong7885@gmail.com; 3 Faculty of Information Technology, Hung Yen University of Technology and Education, Hung Yen 160000, Vietnam

**Keywords:** NILM, complex background, denoising, load identification, GAN, CNN

## Abstract

Non-Intrusive Load Monitoring (NILM) allows load identification of appliances through a single sensor. By using NILM, users can monitor their electricity consumption, which is beneficial for energy efficiency or energy saving. In advance NILM systems, identification of appliances on/off events should be processed instantly. Thus, it is necessary to use an extremely short period signal of appliances to shorten the time delay for users to acquire event information. However, acquiring event information from a short period signal raises another problem. The problem is target load feature to be easily mixed with background load. The more complex the background load has, the noisier the target load occurs. This issue certainly reduces the appliance identification performance. Therefore, we provide a novel methodology that leverages Generative Adversarial Network (GAN) to generate noise distribution of background load then use it to generate a clear target load. We also built a Convolutional Neural Network (CNN) model to identify load based on single load data. Then we use that CNN model to evaluate the target load generated by GAN. The result shows that GAN is powerful to denoise background load across the complex load. It yields a high accuracy of load identification which could reach 92.04%.

## 1. Introduction

Electricity consumption is recently increasing because of the large demand for energy usage at factories, buildings, or residential homes. It may risk the lack of electricity, especially when energy demands are more and more higher in the world. One of the most effective methods to solve this issue is developing a better energy monitoring. Energy monitoring provides information related to the event and power consumption of each appliance. In practice, this monitoring has a positive effect on residents’ behavior. Studies have shown that making users aware of how many power consumed can reduce power consumption by approximately 15% [1,2].

Energy monitoring itself divides into two approaches: Intrusive Load Monitoring (ILM) and Non-Intrusive Load Monitoring (NILM). The number of sensors is a major difference between two concepts. In particular ILM installs at least one sensor for each appliance to monitor the load, while NILM installs one sensor per house [3]. Therefore, NILM is easier and cheaper in physical installation. However, NILM needs a robust approach to monitor the load of each appliance. It is because using one sensor will aggregate every load of appliances in a house. Thus, NILM based approaches are widely attracted more researchers [4].

There are two main objectives in NILM research—energy disaggregation and load identification/ recognition. The differences along with relations between them are listed as follows.

- Energy disaggregation aims to obtain energy consumption for every single appliance, while load identification aims to detect transient-state processes of on/off events [3].
- Energy disaggregation takes entire operation cycle of an appliance (some studies discard event detection (on/off events) and gives a result at the hour level to disaggregate the energy consumption [5]), while load identification takes uncompleted operation cycle, that is the transient-state process of on/off events.
- The main concern of energy disaggregation is approximating power consumption of each appliance in a certain period; thus, the exact on/off time of appliances is ignored. Otherwise, load identification concern about how to acquire appliance operation status [6].
- The approach [7] combines both load identification and energy disaggregation based on unsupervised clustering technique. Recent research [8] proposes both hardware and software for load identification and power consumption monitoring of individual appliances. However, the design system is quite complex. Hardware equipment as well as software are hard to be deployed or maintained in real residential and industrial settings.

In this paper, we concern on load identification problems. Load identification is commonly used in some advanced applications of smart grid for remote household control [5] such as demand response. Demand response identifies the type of connected load and decides whether to switch load off in a situation of reaching peak load consumption. The process of load detection and disaggregation should be done automatically in order to have an easier microgrid set-up [9]. The result of load identification further also benefits for energy disaggregation.

Several studies have been conducted to address the load identification problem. Hidden Markov Model (HMM) was used for automated load identification [9]. Ant colony optimization algorithm for the load identification process was also applied [10]. The authors of Reference [11] used back-propagation for load identification with utilizing power spectrum of transform coefficients (WTCs) to effectively reduce the number of WTCs representing load turning-on/off transient signals. Reference [2] succeeded in identifying which appliance has caused the turning-on event using two-step classification algorithm. The first step is to classify the appliance’s type. And the second step is identify the appliance’s name based on that type. Reference [12] also proposed a method for load identification using turning-on transient state. The author addresses the problem using only a handful of loads whose electric properties often differed significantly from each other. The recent research in Reference [3] proposed a method named Concatenate Convolutional Neural Network regarding load identification. The previous research in References [2,9,10,11] did not take segment data as short signal in load identification, which is not beneficial for advanced NILM system. In contrast, Reference [3] has already concerned advanced implementation which used the segment data from turning-on transient state signal with short period. In the smart grid, some advanced applications need to be done quickly for remote household control, thus acquiring appliance operation status using short transient signal is crucial [3,5].

The authors of Reference [12] used short signal, but they did not consider combined load. In contrast, Reference [3] utilized on both short signal as load identification feature and combined load. However, the author did not focus on denoising background load as noise. The load identification method only relied on similarity and difference of load data. Thus, the result of load identification is obtained in low accuracy for some appliances. When more appliances are switched on within the background load, the overall accuracy of the algorithm is decreased. The reason is when more appliances are simultaneously operating with the background, the more noise is added to the system [2].

In this paper, we aim to solve that issue by assuming background load as noise and generate its noise distribution using Generative Adversarial Network (GAN). We transpose the short signal into a spectrogram image based on Short-Time Fourier Transform (STFT) and denoise this spectrogram image as a computer vision task. Furthermore, GAN has recently been proved successfully in numerous computer vision tasks including denoising for imaging [13]. Thus, GAN is considered as a powerful method to be implemented in generating the noise distribution of target load over complex background load. By using the generated noise, a clean target load can be obtained. After that, the clean target load can be used to enhance load identification. We train Convolutional Neural Network (CNN)-based load identification model using single load data, then we use the trained model to evaluate the generated target load. Our main contributions are described as below:

- We use GAN to generate noise distribution from complex mixed load by empowering the utility of GAN in image denoising issues. The noise distribution is used to process clean target load. The clean target load is used as the input of load identification model.
- We develop CNN-based load identification model and then train it using single load data. After that, we use the trained model to evaluate target load generated by GAN.
- We implement and evaluate our proposed method using public dataset from Laboratory for Innovation and Technology in Embedded Systems, called LIT-dataset [14]. The experiment results show that our proposed method is powerful to generate the target load across the complex load by denosing background load. The proposed method yields a high accuracy of load identification which reaches 92.04%.

The rest of paper is constructed as follows. In Section 2, the related works about load identification in NILM is discussed. In Section 3, the proposed method is explained. In Section 4, the experiment, results, and discussion are provided. Finally, the conclusion is in Section 5.

## 2. Related Work

NILM is designed to monitor several appliances connected to only one electrical circuit [15]. At the beginning, NILM research was mostly related with energy disaggregation issue. Energy disaggregation aims to find which appliances operating in an aggregated load and how many powers consumed by individual appliances. The observed load in energy disaggregation does not concern about its time, it can be an hour or even a day [5]. Recent advanced development of NILM have been attracting many researchers, such as advanced applications in the smart grid which acquires appliance operation status for remote household control [3,5]. For instance, system require to know which appliance is turning-on and how the power is increased. If it is over the limit of available resource, then system will automatically turn it off or household will automatically be informed to take the next action. Load identification aims to find which appliance cause the turning-on event. The observed load in load identification should be short because the control response should be fast.

Load identification (or Load recognition) in NILM refers to classification methods which map input data to a certain load type [10,16]. In load identification, current and/or voltage of an aggregated load which comprise many individual loads is mostly analyzed. Turning-on event is assumed that it is caused by exactly one turning-on appliance [16]. For each turning-on event, features are extracted by using several disaggregation methods and then one load type is predicted. Thus load identification problem can be also simplified as a supervised classification problem.

Transient state is transitional state that appliance goes through when it is switched on, before it reaches its operational steady state. Transient state is mostly utilized as the feature in load identification, instead of steady state. The reason is because steady-state of appliances only comes when transient-state has faded out which consequently waiting is required [17]. Nevertheless, magnitude and phase at odd-numbered harmonics are generated based on Fast Fourier Transform as a novel steady-state feature to enhance load identification [18]. Several studies have already shown that the transient current can be successfully used to identify electric loads [10,12]. References [17,19,20,21] suggest that most of the appliances observed in fields have partially or completely repeatable transient profiles, due to their unique physical characteristic. As a result, transient analysis can be conducted for load identification [22,23].

Some research is being carried out in the field of NILM addressing load identification issue. Hidden Markov Models (HMM) is proposed to identify load for demand response in microgrid [9]. In the research, this method is used to estimate the state-change probabilities of different appliances and recognition is made on this estimation. Hence load identification can be performed, and inessential load can be turned off so that the lack between energy storage and demand can be avoided. The feature that being used are average energy consumption (AEC), edge counts (EC), percentage energy consumption (PEC), taken from power signal with window size in 2 min. Because the feature is taken from 2-min window size, HMM method is considered not effective enough in term of load identification time. Besides, some of appliances have low accuracy (e.g., printer, coffee machine, and computer).

NILM proposed in Reference [10] employs an Ant Colony Optimization (ACO) algorithm to identify the operation status of individual household appliances. The method extracts transient state feature of current load using S-transform (ST) method. The length of transient signal taken based on how long the transient occurred on an appliance. The research can identify which appliances composing a combined load which is an advantage. However, ACO method also has a drawback because the loads that being identified from a combination load, which do not specify when appliance is switched on. Furthermore, the accuracy is also still low which is only 79%. Hence, ACO cannot inform which appliance causing the event and would not be helpful for demands response case.

A power spectrum of the WTC (Wavelet Transform Coefficients) [11] in different scales calculated by Parseval’s theorem is proposed. By using this theorem, the number of transient signal features can be reduced without losing its fidelity. WTC itself contains plenty of information needed for turning-on/off transient signal which may useful for load identification. But, the method requires longer computation time and larger memory requirements. Meanwhile for the load identification, backpropagation neural network is used. WTC has high success rates, but it has some drawbacks. First, the method also takes the transient state length depending on how long the transient occurred. Second, the method does not consider background load and only being experimented in single load.

Reference [2] used a two-step classification algorithm to identify which appliance has caused the event when an appliance switches ON or OFF. Step one: they classify load type whether it is type I or type II using the transient signal in 40 milliseconds. Type I loads are linear nonreactive loads and Type II are nonlinear loads and linear reactive loads. Step two: they classify the appliance (from 15 appliance classes) using steady-state signal from five seconds after each transient and recorded for a further ten seconds, and Naive Bayes algorithm as the classifier. The step one has good accuracy which reaches 0.936. The step two is also good cause it can identify the load across various background loads. However, in terms of time operation it is not efficient because it needs 15 s of transient signal after the transient.

The authors of Reference [12] utilize Support Vector Machine (SVM) for load identification using only turning-on transient state of current feature. The method succeeded to distinguish 18 different fixed speed motors, which is challenging because electric properties barely differed significantly from each other. For each turning-on event, several features were calculated based on the second to sixth period of the current after the turning-on event, which corresponds to the first approximately 20–120 milliseconds after a turning-on event. SVM method achieved F1-score of 97.7%. However, the method only extracted features for each switching event of single load.

The load recognition techniques described above are based on identification of an individual load. Most of them are having issue either in the term of time operation or load combination (load containing background loads). The recent research in Reference [3] can solve those issue by proposing a method called Concatenate Convolutional Neural Network. The author took the feature from turning-on transient state signal with only 7 s of window size. The method can also identify a load even though the load is operating across background load. However, the research did not focus on denoising issue of a target load with considering background load as noises. The load identification method only relied on similarity and difference learning and did not specifically learn background load as the noise. Thus, this method has low accuracy for some types of appliance. Whereas, the requirement of the proposed system is to denoise background load without violate the short-size windows taken as the sample. Therefore, an adopting approach from image denoising domain are utilized to overcome this issue, which are described in the following section.

## 3. Proposed Method

Our proposed idea is inspired from computer vision issue related image denoising. Any load signal can be represented as image by transforming it into spectrogram image. References [24,25,26] used pixel from spectrogram image (transformed from a signal) as the feature of recognition task and then has performed promising result. Because colors distribution of spectrogram image is unique and can represent the feature of each recognition class. Therefore, denoising of image can also be performed based on spectrogram image.

Image denoising itself is a common issue in computer vision and image processing. It is not only a useful low-level image processing tool to provide high-quality image, but also an important preprocessing step for many deep learning problems, including identification. Image denoising aims to recover the clean image $\hat{X}$ from its noisy observation *X* which is contaminated by noise distribution *E* [27].

(1) $$X=\hat{X}+E.$$

Estimating $\hat{X}$ from *X* is an inverse problem and the challenge is how to model the function that is able to generate *E*, so that *X* can be estimated. Many studies have been conducted to estimate *E*. Recently, GAN has commonly used and proven its effectively to solve this task [28,29,30,31,32,33].

In this paper, we propose GAN to denoise the target load from complex background load for addressing the drawbacks associated with the existing NILM approaches surveyed in Section 2. Next, we describe problem definition and framework of proposed method.

### 3.1. Problem Definition

**Figure 1:**
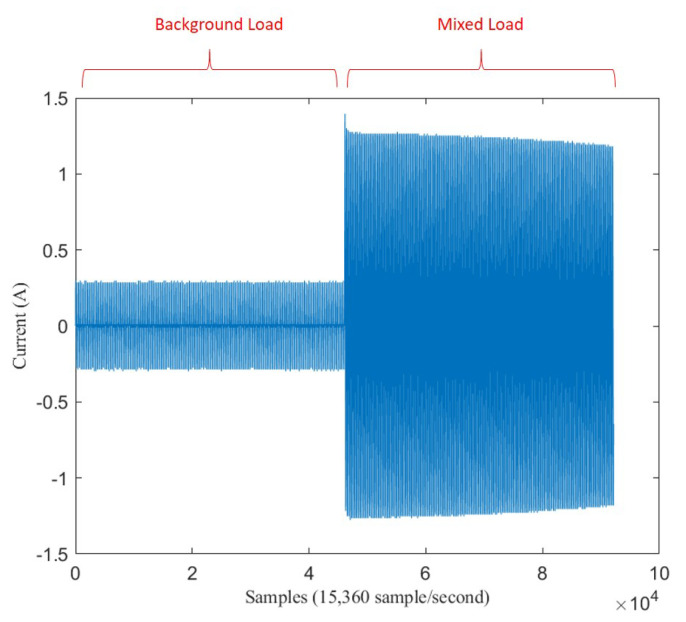
Six seconds of mixed load between LED Lamp and Fan.

As the problem statement, assume a sequence data of electricity signal $X_{1,2,3,\ldots n}$ which has length *n* points with frequency sampling fs. fs defines how many numbers of point in a second so that the length of *X* in second unit is n/fs. Figure 1 shows the example of sequence *X* that has been plotted.

In the middle of sequence, there is a spike signal, called transient state. Transient state happens when an appliance has just switched on. Assume transient state happens at point *m*. This appliance signal which has just switched on is called target load, $T_{m,\ldots ,n}$ with length n−m. The signal before transient state is called background load, $B_{1,2,3,\ldots ,m}$, with length *m*. Background load represents appliances that have already been turned on before transient happens. If there is no other load operating before an appliance turns on, a target load is the same as single load which is expressed $T_{m,\ldots ,n}=S_{m,\ldots ,n}$. But, if there is another load operating before an appliance turning-on, this target load is mixed within background load, which is called mixed load, $M_{m,\ldots ,n}$.

Load identification model learns a mapping from target load *T* to output of appliance class *A* so that function F:T→A. Load identification problem is function F cannot easily identify *A* if *T* is mixed within *B*, forming *M* so that F:M↛A. Therefore, in this paper we attempt to solve this issue by finding a novel method that satisfies G:B,M→T so that F:T→A.

**Figure 2:**
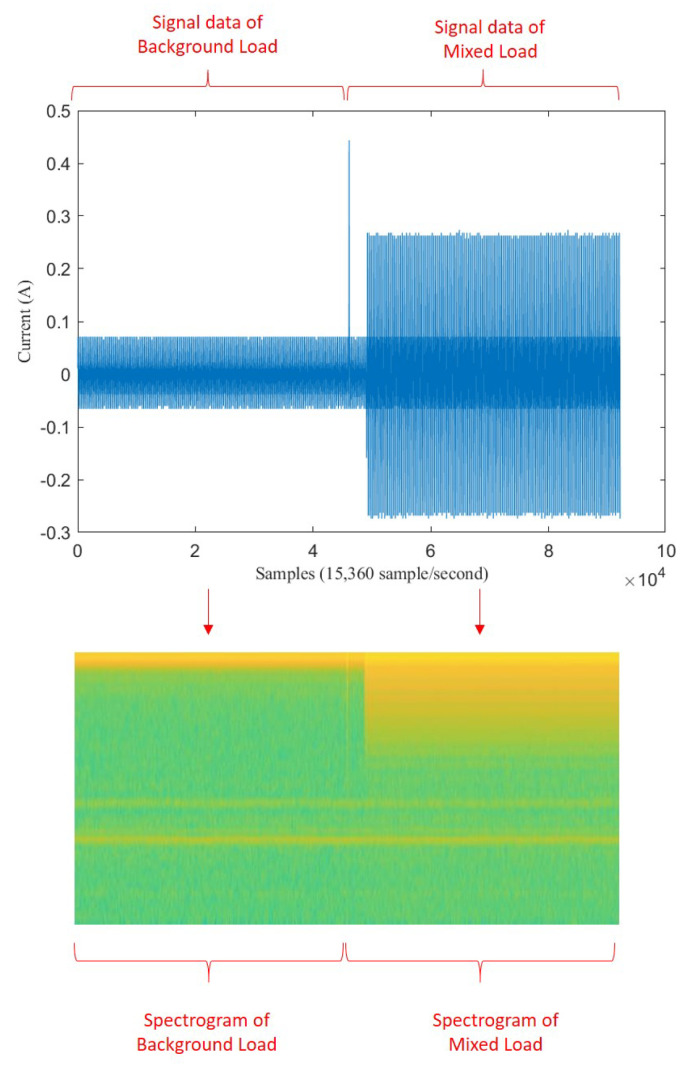
Illustration of transformation from signal to spectrogram.

First, signal $X_{1\ldots n}$ is transformed into an image of spectrogram $S_{\left(1\ldots i,1\ldots j\right)}$ based on STFT. Figure 2 illustrates the transformation from a sequence data of signal into image data of spectrogram.

We assume that background load on spectrogram image $S_{\left(1\ldots i,1\ldots j\right)}$ represents the noise image, target load on spectrogram image $S_{\left(1\ldots i,1\ldots j\right)}$ represents clean image, and mixed load on spectrogram $S_{\left(1\ldots i,1\ldots j\right)}$ represents the noisy image. We express spectrogram of background load as $S_{b(1\ldots i/2,1\ldots j/2)}$, spectrogram of mixed load as $S_{m(i/2\ldots i,j/2\ldots j)}$, and spectrogram of target load as $S_{t(i/2\ldots i,j/2\ldots j)}$. According to Equation (1), we can get:

(2) $$S_{m}=S_{t}+E.$$

In the case where there is no background load, target load $S_{t}$ is same as single load $S_{s}$, expressed as:

(3) $$S_{m}=S_{s}+E.$$

By this way, we use GAN model to generate noise distribution E, based on background load $S_{b}$ and mixed load $S_{m}$, so that:

(4) $$GAN:S_{b},S_{m}\to E.$$

Then referring to Equation (2), we obtain the clean target load by subtracting mixed load with the noise distribution, so that:

(5) $$S_{t}=S_{m}-E.$$

Finally, load identification is performed using that generated target load $S_{t}$ to identify appliance class *A* so that:

(6) $$CNN:S_{t}\to A.$$

### 3.2. Framework of Proposed Method

**Figure 3:**
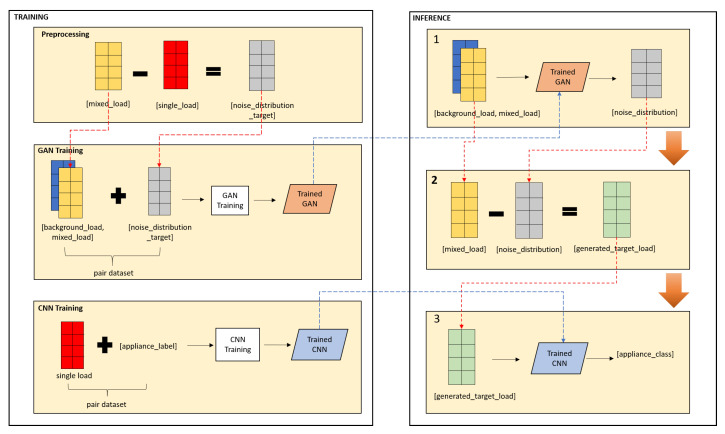
Framework of proposed method.

The proposed idea is illustrated in Figure 3. The proposed method comprises of two general process—training and inferences. Training process aims to build GAN model and CNN model. Inference process is where the load identification implemented. There are three main steps in inference process. Step 1: noise generator model GAN which receives background load and mixed load and output noise distribution, as described in Equation (4). Step 2: target load is calculated based on that noise distribution using Equation (5). Step 3: load identification model CNN which receives generated target load and output appliance class, as described in Equation (6).

The proposed idea includes two model architectures. First model architecture is GAN which is a noise generator model. Second one is CNN as load identification model. Further explanation about these models are described in the next sub sections.

#### 3.2.1. Noise Generator Based on GAN

The proposed noise distribution based on GAN model on load signal is adapted from Pix2Pix GAN by Isola [34]. It is a type of conditional GAN, where the generation of the output image depends on an input or a source image. The Discriminator determines whether the target is a plausible transformation of the source image, based on a source image and the target image provided.

**Figure 4:**
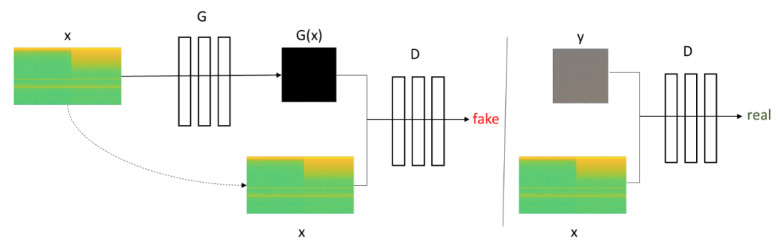
Training of conditional Generative Adversarial Network (GAN).

GAN learns a mapping from random noise vector *z* to output image *y*, G:z→y [34]. In contrast, conditional GANs learn a mapping from observed image *x* and random noise vector *z*, to *y*, G:x,z→y. The generator *G* is trained to produce outputs that cannot be distinguished from “real” images by an adversarially trained Discriminator, *D*, which is trained to do as well as possible at detecting the generator’s “fakes”. This training procedure is illustrated in Figure 4.

In general the objective of GAN is *G* tries to minimize this objective against an adversarial *D* that tries to maximize it, that is, $G^{*}=argmin_{G}max_{D}L_{GAN}\left(G,D\right)$. But in Pix2Pix GAN, the objective of this is a combination between the objective of conditional GAN (Equation (9)) and Loss L1 distance (Equation (8)) as L1 encourages less blurring [34]. It can be seen in Equation (7), where λ is the weight on the L1 objective relative to the GAN objective. This combination is because of the beneficent to mix the GAN objective with a more traditional loss [35]. In this way, the Discriminator’s task remains unchanged, but the generator is tasked to not only fool the Discriminator but also to be near the ground truth output in an L1 sense:

(7) $$G^{*}=argmin_{G}max_{D}L_{GAN}\left(G,D\right)+\lambda L_{L1}\left(G\right),$$

where,

(8) $$L_{L1}\left(G\right)=E_{x,y,z}\left[{∥y-G(x,y)∥}_{1}\right],$$

(9) $$L_{cGAN}\left(G,D\right)=E_{x,y}\left[\log D\left(x,y\right)\right]+E_{x,z}\left[\log\left(1-D\left(x,G\left(x,z\right)\right)\right)\right].$$

**Figure 5:**
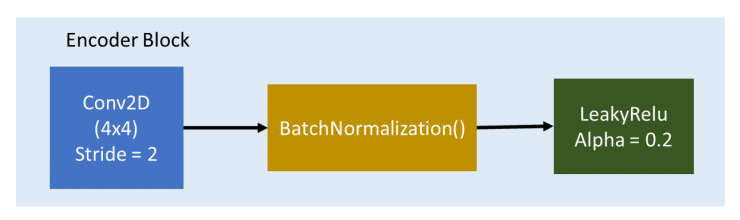
Encoder block.

**Figure 6:**
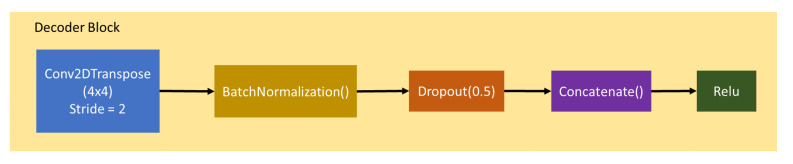
Decoder block.

**Figure 7:**
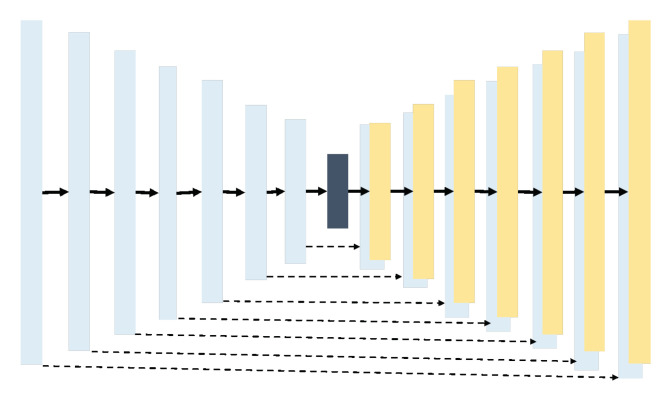
GAN architecture.

The generator architecture consists of 7 encoder blocks and 7 decoder blocks with bottleneck layer that connect the blocks. Encoder Block composes of Con2D, BatchNormalization, and Leaky ReLU (see Figure 5), while Decoder block composes of Conv2Transpose, BatchNormalization, Concatenate layer, and ReLU Activation(see Figure 6). The Bottleneck Layer compose Conv2D and ReLU. It is represented as dark blue color in Figure 7.

The dropout applied on several layers on generator is because the generator simply learned to ignore the noise *z* [34]. Then skip connection was also added. Skip connection is represented as dotted line in Figure 7 that connects encoder block and decoder block. This skip connection is basically the concatenation between encoder output and decoder output, processed by concatenate layer in Decoder block. Skip connection solves bottleneck issue in encoder-decoder network. In such a network, the input is passed through a series of layers that progressively down-sample, until a bottleneck layer, at which point the process is reversed. Such a network requires that all information flow pass through all the layers, including the bottleneck. For many image translation problems, there is a great deal of low-level information shared between the input and output, and shuttling this information directly across the net would be desirable. In other words, skip connection can connect layers in the encoder with corresponding layers in the decoder that have the same sized feature maps.

The input of generator is the concatenate between 3 channels spectrogram image of background load and 3 channels spectrogram image of mixed load, while the output is 3 channels of noise distribution.

**Figure 8:**
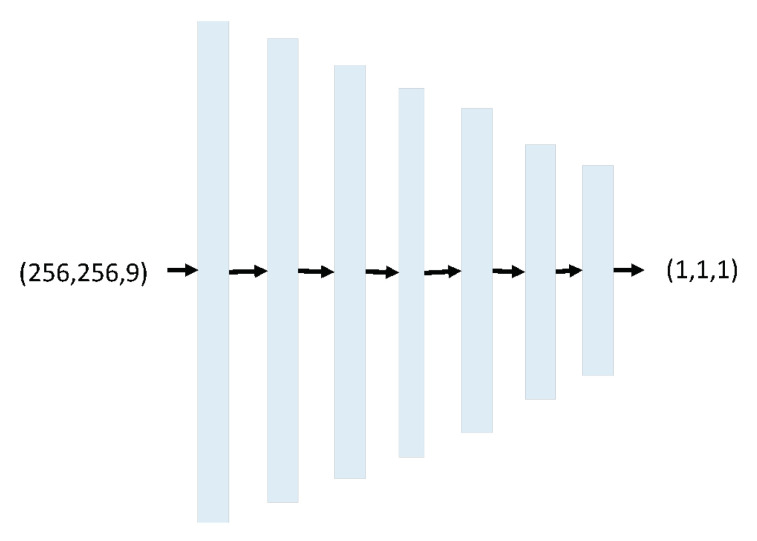
Discriminator architecture.

Discriminator architecture consists of 1 Concatenate layer, 5 Conv2D layers with Leaky ReLU activation, and 1 Conv2D layer with sigmoid activation (see Figure 8).

The loss function of Discriminator is Binary Cross-entropy because basically Discriminator is like common CNN model with two-classes classification task, classifying image input into real or fake. The Cross-entropy function formulated as:

(10) $$L=(\Sigma_{i=1}^{n}y_{i}\log a_{i}+\left(1-y_{i}\right)\log\left(1-a_{i}\right)),$$

where the variable *n* denotes the total number of features for training, variable *a* denotes actual outputs and variable *y* denotes desired outputs.

The input of Discriminator is source image which is the input of Generator, and noise distribution target which is calculated from Equation (3). The output of Discriminator is patch image with size 1 × 1. The concept of patch is designed based on the size of the receptive field, sometimes called the effective receptive field. The receptive field is the relationship between one output activation of the model to an area on the input image. The 1 × 1 output patch means that the Discriminator will classify 1 × 1 patches of the input image as real or fake. We run this Discriminator convolutionally across the image, averaging all responses to provide the ultimate output of the Discriminator *D*. We chose 1 × 1 because smaller PatchGAN has fewer parameters, runs faster, and can be applied to arbitrarily large images [34].

#### 3.2.2. Load Identification Based on CNN

CNN model was used as load identification method. CNN is a branch of artificial neural networks which is inspired by visual cortex of human leverages the concept of convolution and pooling. CNN can extract features without omitting the spatial correlations of the input [11]. Hence this model is suitable to be used for image recognition and image classification.

Our approached CNN took spectrogram of single load as the input of the model. Then this spectrogram was processed through several convolutional layers, pooling layer, and fully connected layers, till output a specific class of appliance type. The final classifier determined the label of the target load through the target feature maps. The loss function was the Cross-entropy function:

(11) $$L=(\Sigma_{i=1}^{c}y_{i}\log Z_{i}+\left(1-y_{i}\right)\log\left(1-Z_{i}\right)),$$

where $y_{i}$ is the *i*th bit of the one-hot label *y* of *C* classes, and $Z_{i}$ is the *i*th element of the network output *Z* with the softmax activation, which can be represented as:

(12) $$Z=softmax(h_{\sigma }\left(S_{t}\right)),$$

where $h_{\sigma }$ is the classifier to map the spectrogram of target load to the dimensional vector, and softmax is the activation function.

**Figure 9:**
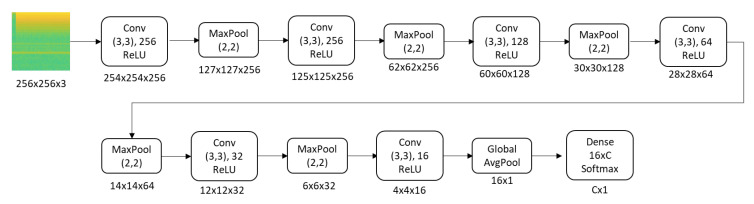
Convolutional Neural Network (CNN) architecture.

Figure 9 presents the detail architecture of the proposed network. There are two parts including, the first part acts as a feature extractor and the second part act as a classifier. The feature extractor part aims to reduce the input size. It consists of 6 convolution layers which comprises of 16 to 256 filter convolution of kernel size 3 × 3 and stride 1, and 5 max pooling layers with size 2 × 2. These layers are followed by a global average-pooling layer to compress the feature map in height and width. The classifier part consists of one fully connected layers with 16 × C dimension (C is the number of classes).

## 4. Experiments, Results, and Discussion

The proposed method was experimented and evaluated using LIT-dataset. This section describes about that in details from dataset, preprocessing, and model implementation.

### 4.1. Dataset

LIT-dataset [14] has 26 types of appliances such as microwave, fan, television, and so on. It provides signals of current and voltage of each appliance with frequency sampling 15.360. It provides binary data showing turning-on event with digit 1 and turning-off event with digit 0. It also provides label of each target load with various scenarios of background load. Some background loads consist of one appliance signal and some others consist of more than one combination of appliance signals. Each scenario has 16 waveforms. This means that each scenario is collected by running 16 rounds over the collect data station, each with a progressive increase triggering-angle (every 22.5 degrees) ranging from 0 to 337.5.

For experiments, the current signals of 9 class appliances were used: LED Lamp, LED Panel, Fume Extractor, Motorola Phone Charger, Fan, Viao Laptop, Drill Speed 2, Microwave Oven ON, and Hairdryer. Those nine types of appliance was chosen because of the variation combination of background load so that the current signals can be used as a set of mixed load data. The mixed load dataset was used to train GAN model. The total data files were 656, with details: 112 files for LED Lamp, 48 files for LED Panel, 80 files for Fume Extractor, 80 files for Motorola Phone Charger, 32 files for Fan, 144 files for Viao Laptop, 64 files for Drill Speed 2, 48 files for Microwave Oven ON, and 48 files for Hairdryer. This data was divided into 70% training data and 30% testing data. Hence the total of training data is 455 and testing data is 201. Table 1 shows the number of mixed load containing each appliances as target load.

In contrast to mixed load size, the set of single load data only consists of 144 data files because each single load in LIT-dataset only contains 16 files. Hence, the single load data used to build the CNN model was split into 90% training data and 10% testing data, which means 126 data files for training (14 files for each appliance) and 18 files for testing (2 files for each appliance). Table 2 shows the number of single load as target load.

**Figure 10:**
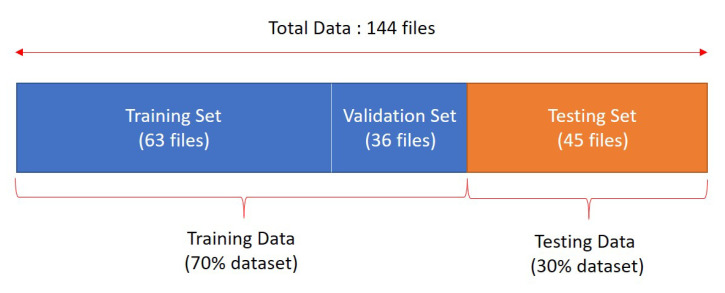
Illustration of splitting data.

The splitting ratio of training and testing data was chosen to be 90:10 after several experiments. At first, the training of CNN was experimented using splitting scenario as Figure 10, which is 70:30 splitting ratio. Because the number of data file is really small, in practice the real training data used was only 63 files (which is only 7 files for each class), 36 files used for validation data (which is only 4 files for each class), and 45 files used for testing (which is only 5 files for each class). In ratio 70:30 splitting data, we found the imbalance problem because the training only used 63 files out of 144 files (training data fraction used was less than 50% of total data). If the number of training data is imbalance, overfitting problem can occur. On the other hand, when we used train-test splitting into 90:10, 108 files was used for training, 18 files was used for validation, and 18 files was used for testing. We found this is more balancing because the training used 108 files out of 144 files (training data fraction used was 75% of total data). Therefore, we chose 90:10 splitting scenario.

### 4.2. Preprocessing

Preprocessing was performed similarly either for mixed load data or single load data, using only current signal dataset. First, binary data representing ON-OFF event that is provided by LIT was read. After that, turning-on index was marked then 3 s before and after transient index was taken. Hence, the length of mixed load signal was 6 s or 92,160 samples, with transient index was located in 46,0810th sample (middle of signal data). But for single load data, only 3 s after the transient index was taken, hence the length of single load signal was 46,080 samples.

**Figure 11:**
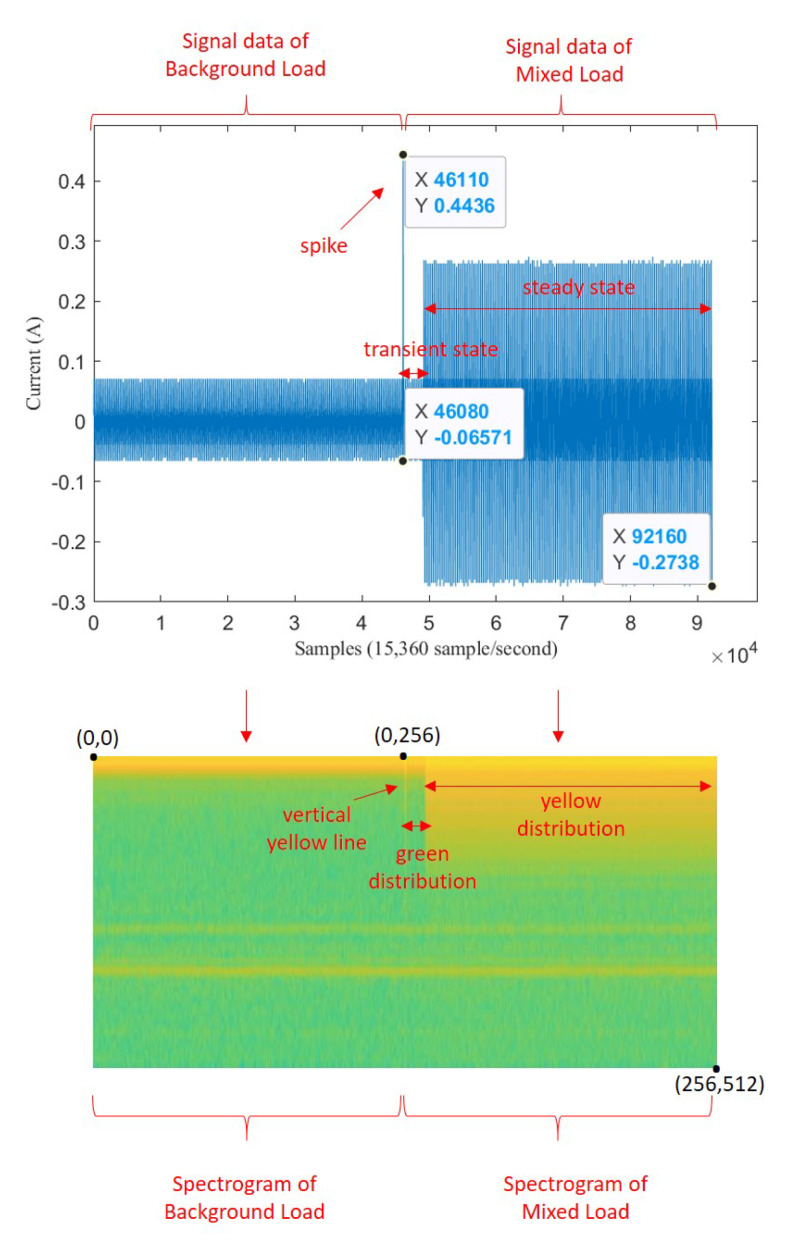
Illustration of transformation from signal to spectrogram on mixed load.

Some signal processing functions in MATLAB tool were used to transform mixed load and single load signals into spectrogram images (*.jpg). For mixed load data, 3 s before the transient index represents background load and 3 s after transient index represents mixed load. The pixel size of spectrogram image was 512 × 256. For illustration, the transformation of mixed load (contains LED Lamp) signal to spectrogram is shown in Figure 11. Since the transient index is located in middle of signal data, we can also see that there is a contrast color in middle of spectrogram image (256th pixel). For instance, when the LED lamp current signal is turning-on, it has short spike line at starting time. It means the vertical line belongs to mixed load. After that, the current signal is going down in short time as transient state, then it is going up again with higher ampere as steady state. In the spectrogram image, in the middle of image there is also one yellow vertical line, then it becomes green for a moment, then it becomes yellow again with more yellow pixel distribution. Therefore, spectrogram image can represent the signal data. The left side represents the spectrogram image of background load ($S_{b}$), while the right side represents the spectrogram image of mixed load ($S_{m}$).

**Figure 12:**
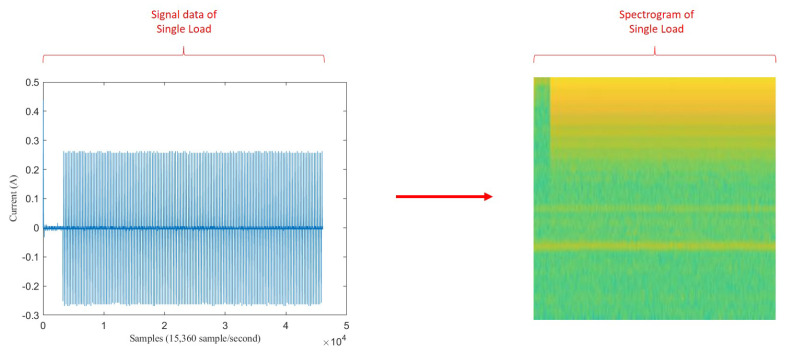
Illustration of transformation from signal to spectrogram on single load.

For single load data, the spectrogram image size was only 256 × 256 because only 3 s signal after the turning-on event was taken. Figure 12 illustrates before-after spectrogram transformation of single load ($S_{s}$). This illustration also used LED lamp signal. Therefore it also has one vertical spike line in at the beginning of signal data, followed by lower current value then higher current value. Correspondingly, it also has one vertical yellow line at the beginning of spectrogram image, followed by short green pixels distribution then long yellow pixels distribution.

A set of noise distribution data was also preprocessed in order to be used in generation training by GAN. In Pix2pix GAN training, a list of paired dataset x,z is needed so that G:x,z→y, where *x* is a list of matrix size (6, 256, 256). The six channels of image size 256 × 256 is the concatenation between 3 channels from background load spectrogram and mixed load spectrogram, expressed as $x=[S_{b},S_{m}]$. While *z* is the noise distribution (*E*) which is used to be the target of generation result (i.e: label image). To generate *E*, Equation (3) was performed.

### 4.3. GAN Experiment

The GAN model was trained using 70% of mixed load dataset (Table 1). The learning optimization was performed using Adam optimizer with learning rate is 0.0002 and parameter beta is 0.5. Adam optimizer was chosen because it has better performance than other stochastic optimization methods [36]. Number of iteration in training was 13,650. The implementation environment comprises Tensorflow and Keras in CPU with specification Intel Core i7 processor, 8GB RAM, and Intel HD Graphics 620. In Keras, the objective function of pix2pix GAN (Equation (7)) was implemented using Cross-entropy and Mean Absolute Error (MAE). Cross-entropy represents the objective of conditional GAN (Equation (9)) and MAE represents the loss L1 (Equation (8)).

**Figure 13:**
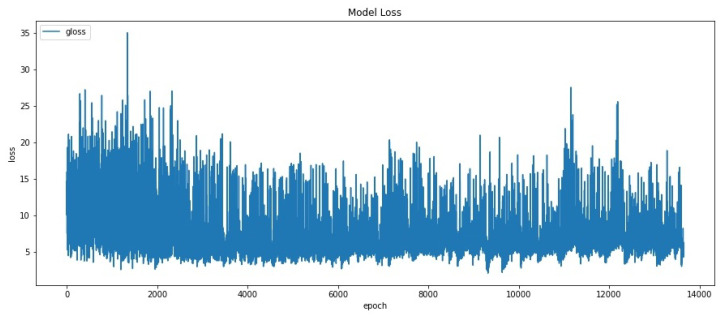
GAN Training Curve based on GAN loss.

The pix2pix GAN objective is represented in term gloss. This gloss is formulated by $G^{*}$ in Equation (7). GAN training curve based on gloss can be seen in Figure 13. Based on the graph, gloss is erratic early in the run before stabilizing after iteration reaches 4000. After 4000th iteration, loss range value remains stable between 5 to 15. The generator is expected to produce its highest quality images during this period of stability.

**Figure 14:**
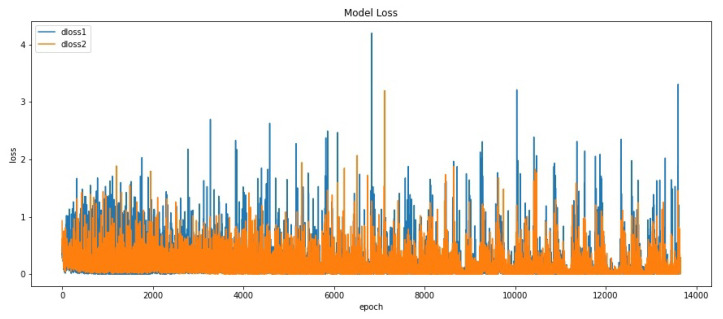
GAN Training Curve based on discriminate loss.

Discriminator loss was implemented using Cross-entropy (see Equation (10)). We plotted two types of Discriminator loss in order to easily compare the performance of Discriminator in discriminating real images and discriminating fake images. Variable dloss1 represents Discriminator loss in discriminating real images. Variable dloss2 represents Discriminator loss in discriminating fake images. Figure 14 shows the plot of dloss1 and dloss2. Based on the figure, it can be seen that dloss2 range values remains lower than dloss1 range values at the beginning. This result shows the generator is poor at generating examples in some consistent way that makes it easy for the Discriminator to identify the fake images. But starting from iteration 9000, dloss1 and dloss2 are the same, which means the Discriminator is fair enough to identify real samples or fake samples (i.e., equilibrium has reached). By this way, we concluded that if the generator model is capable of generating plausible images, then the expectation is those images would have been generated after iteration 9000. Especially, the point where dloss1 and dloss2 are nearly the same (around iteration 12,000 to 13,000).

The trained GAN model was then used to generate noise distribution and process it into clean target loads. It used 30% of mixed load dataset as we mentioned in Section 4.1, which means this data is unseen in the GAN training process. As an example of its result, some generated target load spectrograms of LED Lamp are presented in Table 3. Although the background loads are different, the trained GAN model generated suitable target load for each class. In other words, all the generated target loads of LED lamps are similar to single load of LED lamp, even though various background loads were used (such as Microwave, Soldering Station, Resistor, and others). This observation shows that our GAN has succeeded to denoise the background load so that target load generated is similar to single load. To validate the performance of those generated target loads, a CNN model was trained using single load, and the CNN model was used to test those generated target loads. The details are explained in the next subsection.

### 4.4. CNN Experiment

CNN model was trained using single load data (Table 2). It was also implemented using Tensorflow and Keras in CPU with specification Intel Core i7 processor, 8 GB RAM, and Intel HD Graphics 620. In details, the CNN model was trained only using Adam optimizer with learning rate 0.0001. Adam optimizer was chosen because it can reach the convergence earlier than other stochastic optimization methods [36]. Categorical Cross-entropy was chosen as loss function in our CNN model because load identification is multiclass classification task. Several experiments have been conducted by adjusting parameters including number of epoch, batch size, and train-test dataset splitting ratio. However, setting of 180 epochs and 16 batch size numbers can get the best performance accuracy. We have experimented using higher number than 180 epochs, but the accuracy of training process shows that function is too closely fit to the set of data points, thus we performed early stop by choosing 180 epochs. We have also experimented using different batch size number than 16. However, the CNN model resulted overfitting.

**Figure 15:**
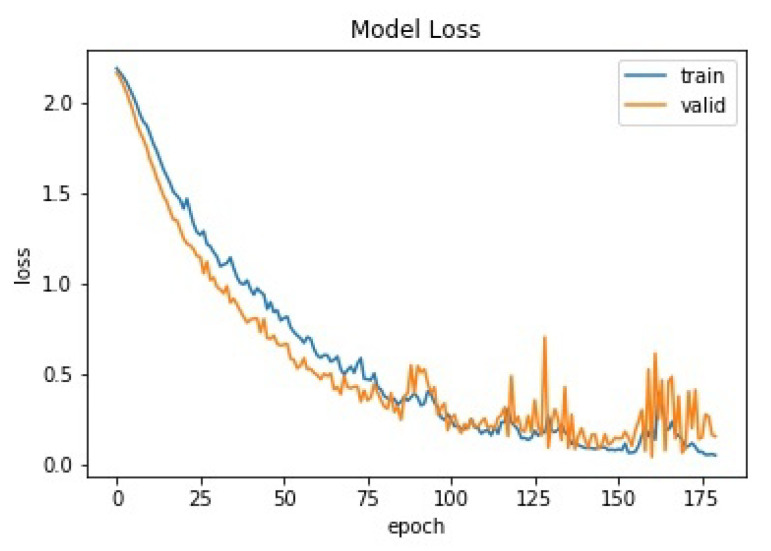
CNN training and validating curves based on loss.

The training result can be seen in Figure 15.

At 180th epoch the loss value for training set is 0.05 and the loss value for validation set is 0.15. And the testing with single load shows accuracy 88.89%, the details can be seen in Table 4. The table result shows most of appliances obtained 100% accuracy. There were only 2 appliances had 50% accuracy, Fume Extractor and Fan. This is because one of the testing data of those 2 appliances was miss-classified. As we mentioned in Section 4.1, single load data that being used for testing were only 2 files for each class. It means if all of those 2 files correct, the accuracy is 100%. If only one of those 2 files correct, the accuracy is 50%.

The CNN trained model was then used to infer target load generated by GAN. Notice that the generated target load used mixed load dataset as the input. The average accuracy of CNN inference is 92.04% along with details accuracy of each class shown in Table 5. The accuracy metric used is $(\Sigma_{idx=1}^{T_{ot}}X_{idx}/T_{ot})\times 100$, where $T_{ot}$ is total testing data of each appliance. Based on the result, it can be seen that almost all classes have high accuracy. Only Fan and Fume extractors have lower accuracy which are 60% and 75%, respectively.

The generated target load of Fume extractor performed by GAN can be seen in Table 6, while the Fan can be seen in Table 7.

The shown result table above illustrates the single load of both appliance and their generated target load are having not significant different. But, based on the testing result of CNN model using single load, both appliances have low accuracy, which are only 50% (see Table 4). In other words, the reason of low accuracy of our proposed method towards Fan and Fume extractor is likely because of the CNN model itself, not the GAN model.

**Figure 16:**
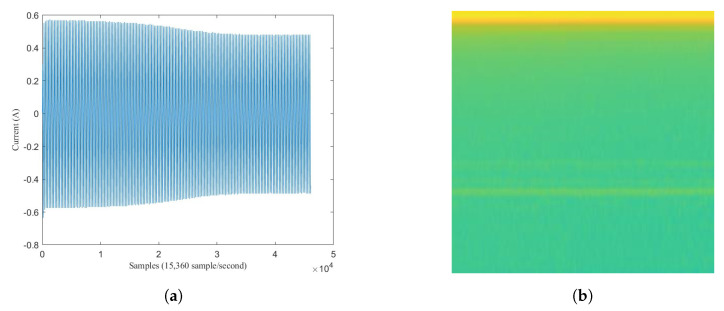
Fume Extractor: (**a**) load signal; (**b**) spectrogram image.

**Figure 17:**
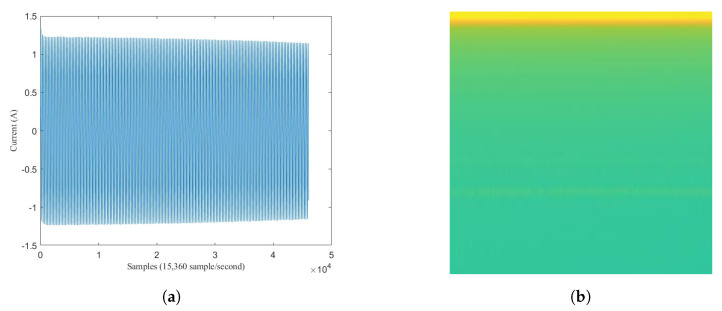
Fan: (**a**) load signal; (**b**) spectrogram image.

Moreover, the signal load of Fan and Fume Extractor have seem to be similar pattern in the transient length and the fluctuation shape. This causes the generated spectrograms on both appliances look like same. In particular, the similar of transient segment extracted before and after 3 s can be seen in Figure 16a for Fume extractor and Figure 17a for Fan. Furthermore, Figure 16b and Figure 17b show spectrogram results almost same. This is a main reason to make the CNN model decrease accuracy performance in case Fan and Fume Extractor which have similar spectrogram images.

### 4.5. Discussion

To confirm our proposed method, we re-implemented Concatenate Convolutional Neural Network (concatenate-CNN) by Reference [3] on the same LIT-dataset dataset. In particular, concatenate-CNN was re-implemented using the same set of mixed load data, either training set or testing set, as being used by GAN model. The training and testing were also performed using same device and same environment setting as our proposed method. The obtained results show that their concatenate-CNN has accuracy of 69.30%, while our method has accuracy of 92.04%.

**Figure 18:**
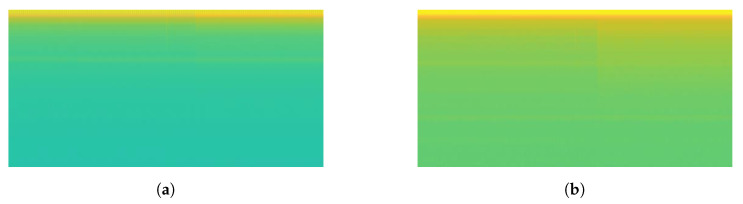
The spectrogram results of mixed load data containing LED Lamp: (**a**) Load Combination of Hair Dryer Super-2 and LED Lamp; (**b**) Load Combination of Hair Dryer Eleganza and LED Lamp.

**Figure 19:**
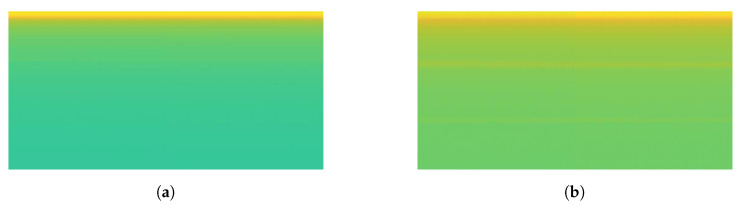
The spectrogram results of mixed load data containing Fume Extractor: (**a**) Load Combination of Oil Heater Power-1, Hair Dryer Super-2, and Fume Extractor; (**b**) Load Combination of Hair Dryer Super-1 and Fume Extractor.

**Figure 20:**
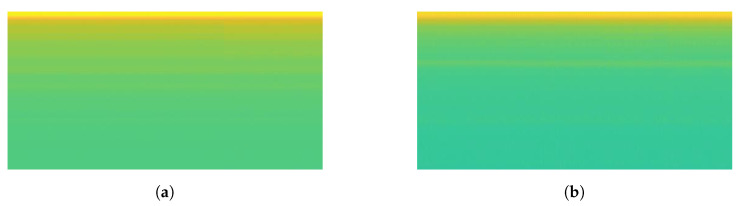
The spectrogram results of mixed load data containing Motorola Phone Charger: (**a**) Load Combination of LED Panel, Oil Heater Power-1, and Motorola Phone Charger; (**b**) Load Combination of Vaio Laptop, Hair Dryer Super-1, and Motorola Phone Charger.

**Figure 21:**
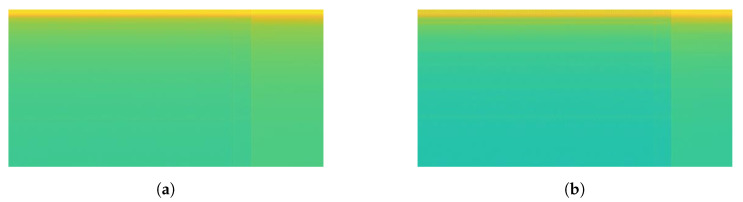
The spectrogram results of mixed load data containing Microwave Oven ON: (**a**) Load Combination of Oil Heater Power-2, Fume Extractor, and Microwave Oven ON; (**b**) Load Combination of LED Panel, Hair Dryer Parlux, and Microwave Oven ON.

Details comparison results is shown in Table 8. The comparison result depicts 7 classes of the proposed method have higher accuracy than concatenate-CNN, while 2 classes (Hair Dryer and Viao Laptop) have absolutely accuracy of 100%. From those 7 classes, there are four classes which contains significantly improved of accuracy by our proposed method. Those are LED Panel, Fume Extractor, Motorola Phone Charger, and Microwave Oven ON with 53.33%, 33.33%, 50%, and 40% increasing percentage, respectively. If we take a look at mixed load dataset, most of the spectrogram of those four appliances consist of background load and mixed load that nearly the same. Some mixed load spectrograms’ examples of those four appliances can be seen in Figure 18, Figure 19, Figure 20 and Figure 21. Thus, concatenate-CNN had lower accuracy for these four appliances. Basically, concatenate-CNN works by calculating the difference and the similarity between background load and mixed load spectrogram. Hence, when the load identification model of concatenate-CNN receives the similar input of background load and mixed load spectrograms, this model decrease the classification performance. Meanwhile, our proposed GAN model has learned the noise distribution of load during training. Thus, clean target load can be extracted using that noise distribution regardless the similarity of background load and mixed load. In summary, the our proposed method has significant performance over the state-of-art.

However, our research has some drawbacks. First drawback is the availability of public dataset that is suitable for experiments in the proposed method is limited. In Pix2pix GAN training, a pair dataset of input and target is needed. In this case, the method requires a dataset of mixed load and single load. Unfortunately, the availability of mixed load as well as single load in public dataset is quite rare. Several public dataset, such as UKDale, REDD, or BLUED, only provide mixed load dataset, while public dataset such as PLAID, WHITED, or COOLL only provide single load dataset [37]. On the other hand, LIT-dataset provides both mixed load and single load data. This is a reason why we used LIT-Dataset in our experiment. However, LIT-dataset itself actually has several limitations. First one is because of the file number of appliances for single load data is small. Second one is the raw data only able to be preprocessed in Matlab. Because the author only provided the access library in MatLab for processing raw data. Therefore, in the future work, our proposed method should be evaluated by applying on other public dataset that contain both single and mix loads. Second drawback is our CNN model has low accuracy on appliances that have similar pattern in term of transient length or fluctuation shape. Therefore, to solve this problem, in the future work we should make robust feature by finding more useful feature and then combine with our proposed method or improve the CNN-based classification model architecture. Beside that, the CNN model also needs to be confirmed using bigger size of single load dataset. The lack of dataset can be solved by collecting the data individually in lab or by performing data augmentation on signal dataset.

## 5. Conclusions

Load identification task aims to identify the type of turning-on appliance load. Load identification has crucial noising issues if the appliance load across complex background load, especially in a case of the short period load. In this paper, GAN is proposed to address the denoising problem of load identification in NILM. Our method solves the denoising problem by generating the noise distribution with GAN and then using this noise to calculate the clean generated target load. The generated target load is inferred using a classification method CNN. This CNN-based classification model already trained using single load data (clean target load) to classify appliances load.

However, the GAN training requires a paired dataset containing both clean target load data and mixed load, which is not many public datasets providing both data types, except LIT-dataset. Therefore, in this work, we evaluate our proposed method on LIT-dataset. The average accuracy achieves 92.04% on this dataset. Also, the proposed method is compared to previous research by re-implementing the method on the same dataset as well as the programming environment. The comparison result has shown significant improvement by 22.74% in identifying several appliances’ mixed loads, especially the complex loads that make spectrogram of background load and mixed load similar. This is because the trained GAN model has learned the noise distribution of load based on the training dataset in our proposed method. Thus, a clean target load can be extracted using that noise distribution regardless of the similarity of background load and mixed load.

## Figures and Tables

**Table 1:** Number of mixed load data containing target load.

Mixed Load	Number of Data
LED Lamp	112
LED Panel	48
Fume Extractor	80
Motorola Phone Charger	80
Fan	32
Viao Laptop	144
Drill Speed 2	64
Microwave Oven ON	48
Hairdryer	48
**Total Data**	**656**

**Table 2:** Number of single load data.

Single Load	Number of Data
LED Lamp	16
LED Panel	16
Fume Extractor	16
Motorola Phone Charger	16
Fan	16
Viao Laptop	16
Drill Speed 2	16
Microwave Oven ON	16
Hairdryer	16
**Total Data**	**144**

**Table 3:** Several results of GAN inference with LED Lamp as target load.

Sequence of Loads	Source Image	Single Load of LED Lamp	Generated Target Load of LED Lamp
**Background load:** Microwave; **Target load:** LED Lamp	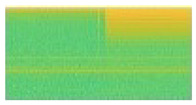	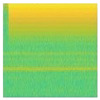	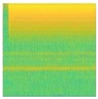
**Background load:** Soldering Station; **Target load:** LED Lamp	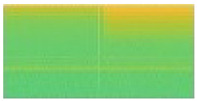	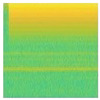	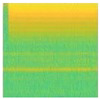
**Background load:** Resistor; **Target load:** LED Lamp	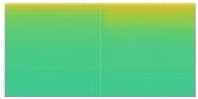	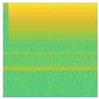	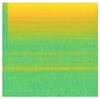
**Background load:** Oil heater and Fan; **Target load:** LED Lamp	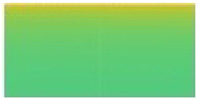	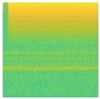	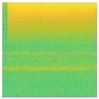

**Table 4:** Accuracy of CNN testing result using single load.

Class	Accuracy (%)
LED Lamp	100
LED Panel	100
Fume Extractor	50
Motorola Phone Charger	100
Fan	50
Viao Laptop	100
Drill Speed 2	100
Microwave Oven ON	100
Hairdryer	100
**AVERAGE ACCURACY**	**88.89**

**Table 5:** Inference accuracy of target load generated using GAN.

Class	Accuracy (%)
LED Lamp	100
LED Panel	100
Fume Extractor	75
Motorola Phone Charger	100
Fan	60
Viao Laptop	100
Drill Speed 2	100
Microwave Oven ON	93.33
Hairdryer	100
**AVERAGE ACCURACY**	**92.04**

**Table 6:** Several results of GAN inference with Fume extractor as target load.

Sequence of Loads	Source Image	Single Load of Fume Extractor	Generated Target Load of Fume Extractor
**Background load:** Oil Heater Power 1 and Hair Dryer Super 4.0—Heater 2; **Target load:** Fume Extractor	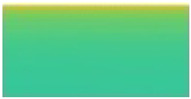	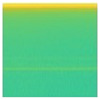	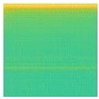
**Background load:** Oil Heater Power 2; **Target load:** Fume Extractor	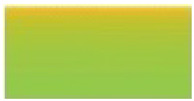	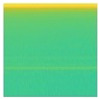	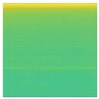
**Background load:** Microwave Oven ON; **Target load:** Fume Extractor	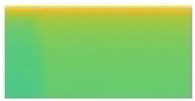	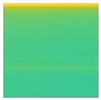	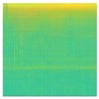
**Background load:** Hair Dryer; **Target load:** Fume Extractor	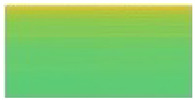	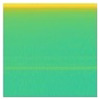	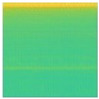

**Table 7:** Several results of GAN inference with Fan as target load.

Sequence of Loads	Source Image	Single Load of Fan	Generated Target Load of Fan
**Background load:** LED Lamp; **Target load:** Fan	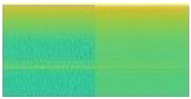	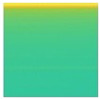	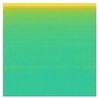
**Background load:** Oil Heater Power 1; **Target load:** Fan	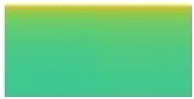	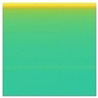	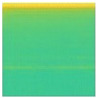

**Table 8:** Comparison result.

Class	Accuracy of Proposed Method (%)	Accuracy of Concatenate-CNN (%)	Improved Accuracy (%)
LED Lamp	100	97.06	2.94
LED Panel	100	46.67	53.33
Fume Extractor	75	41.67	33.33
Motorola Phone Charger	100	50	50
Fan	60	50	10
Viao Laptop	100	100	0
Drill Speed 2	100	85	15
Microwave Oven ON	93.33	53.33	40
Hairdryer	100	100	0
**AVERAGE ACCURACY**	**92.04**	**69.30**	**22.74**
